# The prevalence and risk factors of dental disease found in 100 miniature horses

**DOI:** 10.3389/fvets.2023.1239809

**Published:** 2023-11-30

**Authors:** Tracy Tinsley, Callie Fogle, Elaine Means, James Robertston

**Affiliations:** ^1^Elite Equine Mobile Dentistry, Holly Springs, NC, United States; ^2^Department of Clinical Sciences, North Carolina State University College of Veterinary Medicine, Raleigh, NC, United States; ^3^Elaine Means Mobile Equine Dentistry, Sanford, NC, United States; ^4^Office of Research, North Carolina State University College of Veterinary Medicine, Raleigh, NC, United States

**Keywords:** equine dentistry, miniature horse, dental disease, prevalence, hypodontia

## Abstract

**Introduction:**

Dental disease is a common condition affecting horses. Its prevalence and characteristics among most of the common breeds of horses and donkeys have been investigated and described in the literature, but information about the prevalence and etiology of dental disease of miniature horses is sparse.

**Methods:**

To determine the prevalence and characteristics of dental disease of miniature horses, we performed oral and dental radiographic examinations on 100 miniature horses. The findings of these examinations were analyzed to determine the prevalence of dental disease and its correlation to age, sex, weight, body condition score, height at the withers, head length and head width. Older horses had a higher prevalence of dental disease, diastemata and crown elongations.

**Results:**

The most common dental diseases detected in this population were crown elongation, oral mucosal ulceration, diastemata, class 1 malocclusion and hypodontia. Horses with a high body condition score had an increased likelihood of having a class 1 malocclusion. Horses with wider heads had a higher prevalence of dental disease and class 1 malocclusions.

**Conclusion:**

Frequent oral examinations, starting at an early age, should be prioritized as a part of miniature horse preventive health care to decrease morbidity and slow progression of dental disease.

## 1 Introduction

Miniature horses are a breed of horse that dates to 17th century Europe where they were used to work inside mines (1). Since the 1930s, they have been bred for use as pets, exhibition, novelty, and research (1). There are currently two registries for miniature horses in the United States, the American Miniature Horse Association (AMHA) and The American Miniature Horse Registry (AMHR). There are 200,000 registered horses with the AMHA (1) and the AMHR reports to register 10,000 horses per year (2). Miniature horses are described in textbooks to have a high prevalence of dental disease and unique diseases related to their small stature and disproportionally large teeth, but there is only anecdotal data to support these statements (3). Clauss et al. (4). found that the size of the teeth of horses bred for small stature does not scale proportionally to the size of the animal and their skull. Heck et al. (5) showed adult miniature horses were found to have skull characteristics of juvenile non-miniature horses. These findings suggest there are differences between the miniature horse breeds and their larger relatives that could affect the characteristics and prevalence of dental disease in this population. Numerous studies have described the prevalence and characteristics of dental disease of non-miniature horses (6–10), but the authors were unable to find any published data describing the frequency and types of dental disease in miniature horses. The goal of this study was to perform a cross-sectional analysis of dental diseases of miniature horses and identify any risk factors for their presence. The hypothesis was that miniature horses have a high prevalence of dental disease and malocclusion. It is hoped that the information provided by this study will enable veterinary practitioners to better recognize dental abnormalities that affect miniature horses. Early detection of disease can lead to early treatment and decrease morbidity in this population.

## 2 Materials and methods

Two of the authors (TT and EM) performed all oral examinations and radiographs between January and May 2022 on 100 privately-owned miniature horses. This study was submitted to North Carolina State University’s College of Veterinary Medicine Institutional Animal Care and Use Committee (IACUC) to determine if the study required oversight and approval. This committee determined that an IACUC review was not necessary as all procedures being performed were observational and covered under the patient client relationship. Signed, informed consent was obtained from each owner for the inclusion of their animals in this study.

Height at the withers, head length, head width, body weight (determined by using a weight tape^1^) and body condition scores were recorded for each horse. Age was determined from registration papers for all but nine horses, the owners of which were unable to provide registration papers. The age of these nine horses was determined by information provided by the owner coupled with examination of the horse’s dentition. The height at the last mane hair is traditionally used to determine height in miniature horses for the purpose of registration (maximum height of 38 inches) (1). In this study wither height was used to determine height of the horse as it was repeatable. The length of the head was measured by using a soft measuring tape from the rostral border of the poll to the rostral border of the alar cartilage. The width of the head was measured from the center of the right mandibular condyle across the widest portion of the head to the center of the left mandibular condyle.

**Figure 1:**
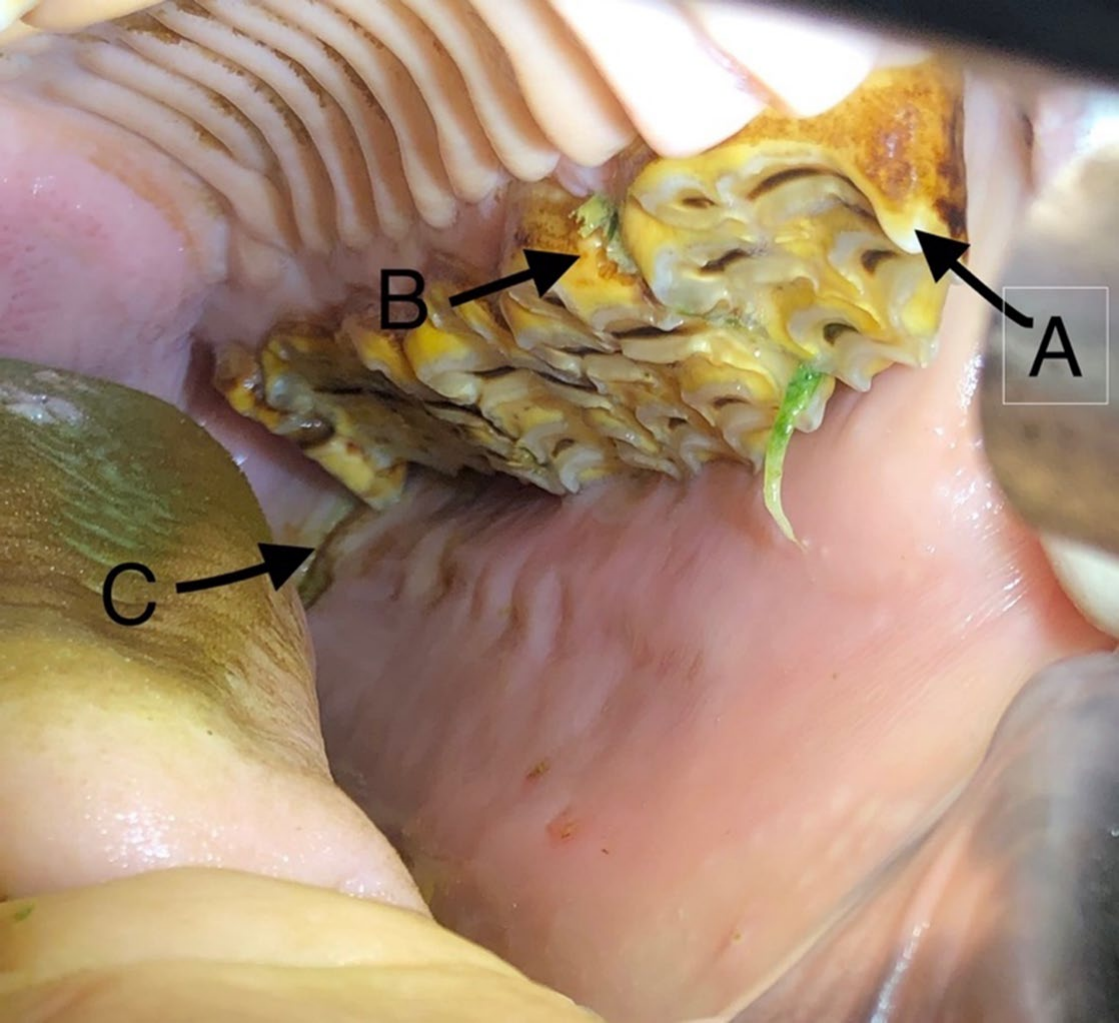
Photograph of a 13 years old miniature mare with (A) mesially elongated 206 crown, (B) valve diastema and (C) buccal oral mucosal ulceration adjacent to a 211 mesial crown elongation.

**Figure 2:**
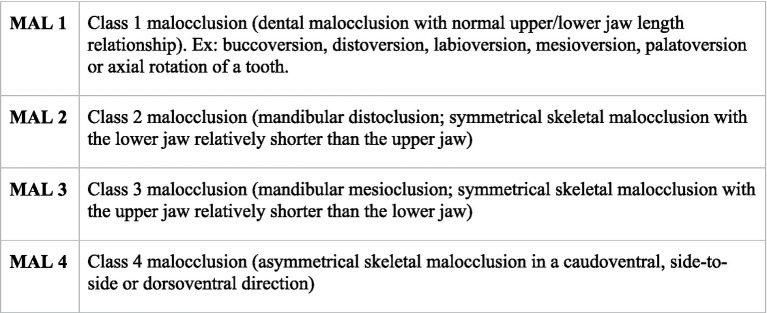
Classification system used for evaluating skeletal and dental malocclusion (11).

After receiving a physical examination, the horses were sedated with a combination of xylazine^2^ (0.5–1 mg/kg), detomidine^3^ (0.02–0.04 mg/kg) and butorphanol^4^ (0.001 mg/kg) intravenously. Dosing was based on the size and temperament of the horse. The oral cavity was irrigated to remove feed and examined. A complete oral examination was performed using a full mouth speculum, a head light and a 45-degree,1 cm bariatric laparoscope^5^ attached by a c-mount adaptor to a modified GoPro camera.^6^ All oroscopic examinations were video recorded and charted on a standard dental chart using nomenclature recommended by the American Veterinary Dental College (11). All disorders of specific cheek teeth and incisors, skeletal malocclusions, injuries to oral soft tissues (buccal/lingual ulceration) and visual or palpable abnormalities of the head were documented. The following abnormalities were identified and classified as follows: class 1 malocclusion (MAL1), class 2 malocclusion (MAL2), class 3 malocclusion (MAL3), class 4 malocclusion (MAL4), valve diastema (D/V) (no open diastemata were identified), oral mucosal ulcerations(C/L), missing tooth, unerupted tooth (T/U), fractured tooth (T/FX), crown elongation (commonly termed dental overgrowths) (T/EL/CC), supernumerary tooth (T/SN), persistent deciduous tooth (DT/P) and dysplastic tooth (11). Figure 1 shows an example of crown elongations, diastema and buccal ulceration in a 13 years old miniature horse. The malocclusion classifications are described in Figure 2. The occlusion at the incisors was used to determine the presence or absence of skeletal malocclusions (MAL2, MAL3, MAL 4) in this study as this is how the breed registries evaluate for this abnormality. A single tooth that was not in proper alignment (displaced or rotated) was categorized as a MAL1. The presence of dental malocclusion at the level of the cheek teeth (for example 06/11 crown elongations) were not categorized as skeletal malocclusions if there was neutral occlusion at the incisors because the variability of the size of the physiologic diastema can create mismatch of the cheek teeth without mismatch of the mandible and maxilla length. Horses were allowed to have both a skeletal malocclusion (MAL2, MAL3, and MAL4) concurrently with a dental malocclusion (MAL1) to state the prevalence of each most accurately in the population.

The presence of sharp enamel points was not documented as dental disease because it is a normal feature of equine dentition.

Six open-mouth, extra-oral digital radiographs of the skull of each horse were obtained by using a digital radiology system.^7^ The views obtained were LeD35-RtVO, RtD35-LeVO, LeV60-RtDO, RtV60-LDO, D-V, Le-Rt latero-lateral. These radiographs were used to clarify and confirm findings of the oral examination.

### 2.1 Statistical analysis

The data from the examination forms were compiled into an Excel spreadsheet. The horses were divided into three categories according to age: juvenile, < 4 years old; adult, 4–15 years old; and geriatric >15 years old. Descriptive statistical methods were used to describe the data. Spearman correlations were used to examine the presence of associations between patient metrics and the dental diseases identified. The presence of each type of disease was considered to be a abnormality. The presence of the same abnormality affecting multiple teeth was considered to be one abnormality, rather than each affected tooth having a abnormality.

Patterns of affected teeth were analyzed with *p*-values testing for divergence from a uniform distribution via Monte-Carlo simulated multinomial testing.

All analyses were conducted in R version 4.2.2 (12).

## 3 Results

Of the 100 miniature horses examined, 69 were mares, 20 were stallions and 11 were geldings. Thirty-two were juvenile horses, 54 were adult and 14 were geriatric. Horses were a median of 8.58 years (range 0.4–40); mean 8.25 ± 7.41 years. Body weight was a median of 122.5 kg (range 37.37–177.3 kg); median 122.5 kg; mean 118.22 kg; standard deviation 33.31 kg. Body condition scores were a median of 5 (range 2–9); mean 5.25; standard deviation of 1.11. Wither height was a median of 93.98 cm (range 73.66–111.76 cm); mean 92.97 cm; standard deviation 9.36 cm. Head length was a median of 37.8 cm (range 31.75–43.18 cm); mean 37.24 cm; standard deviation 3.0 cm. Head width was a median of 22.86 cm (range 17.78–26.67 cm); mean 22.48 cm; standard deviation 1.95 cm. Ninety-five percent of the horses had dental disease, and of these 16% had one disease, 29%, 24%, 17%, and 9% had two, three, four or five concurrent diseases, respectively.

**Figure 3:**
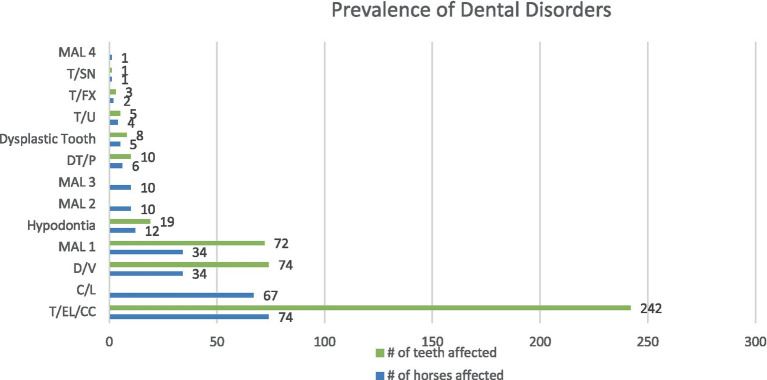
The number of horses and the number of teeth affected by the dental disorders identified in this population. Class 4 malocclusion (MAL4), supernumerary tooth (T/SN), fractured tooth (T/FX), unerupted tooth (T/U), dysplastic tooth, persistent deciduous tooth (DT/P), class 3 malocclusion (MAL3), class 2 malocclusion (MAL2), hypodontia, class 1 malocclusion (MAL1), valve diastemata (D/V), oral mucosal ulceration (C/L) and crown elongations (T/EL/CC).

**Figure 4:**
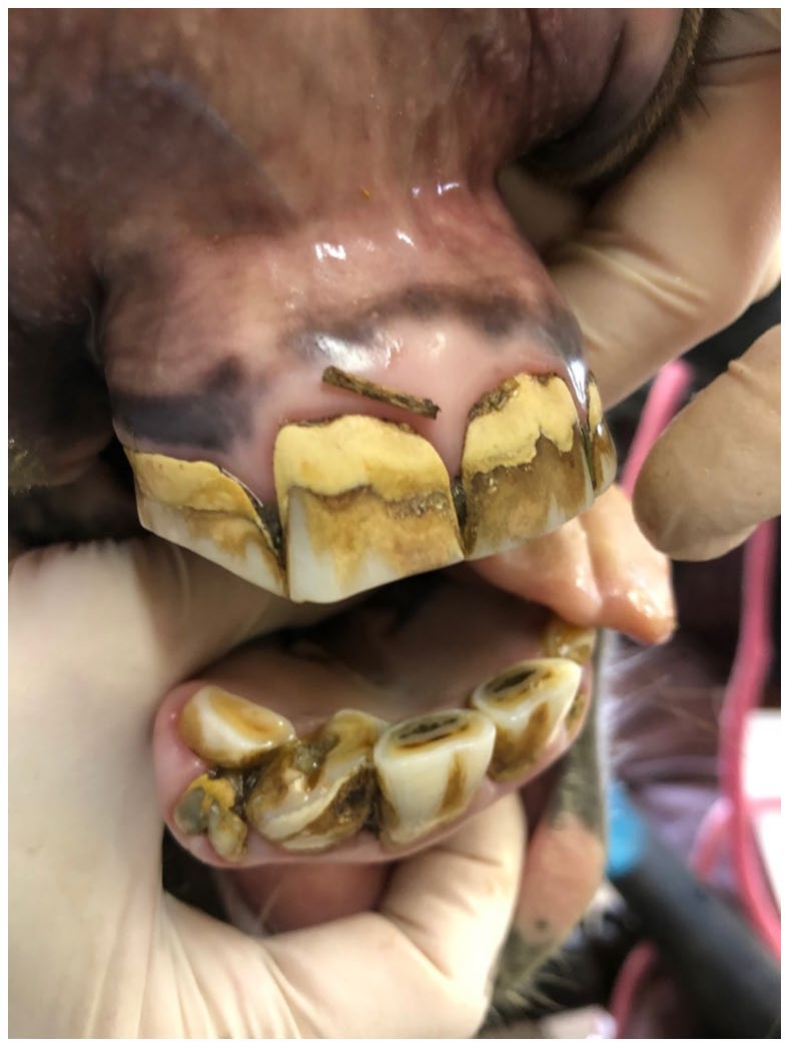
Photograph of a 9 years old miniature mare with axially rotated 402, retained deciduous tooth 803.

**Figure 5:**
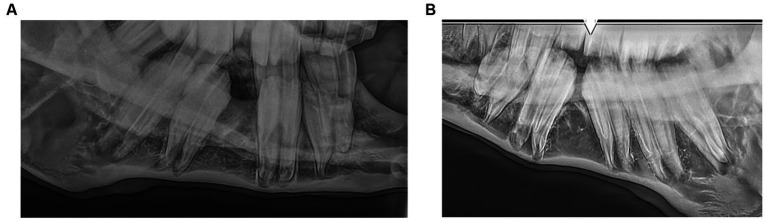
**(A)** LeV60-RtDO and **(B)** RtV60-LeDO radiographs of an 8.5 years-old miniature horse showing hypodontia of teeth 308, 408 and 411.

The most common disease noted was crown elongation followed by oral mucosal ulceration, diastemata, class 1 malocclusion and hypodontia. Figure 3 illustrates the prevalence of these diseases. Crown elongations were identified in 74% of horses examined and affected 242 teeth. The Triadan 06 position was the most frequently affected (115 teeth) followed by the Triadan 11 (47 teeth) and Triadan 08 (32 teeth). The remaining 48 elongated teeth included the Triadan 07s, 09s and 10s and incisors. Ulceration of the buccal or lingual mucosa was identified in 67% of horses (Figure 1). Valve diastemata were identified in 34 horses (34%) with 70 valve diastemata documented. Of these, 19 were identified at the Triadan 09–10 position, 18 at the 08–09 position, 16 at the 10–11 position, 13 at the 06–07 position and 4 at the 07–08 position. Thirty-four horses (34%) were diagnosed with class 1 malocclusion (MAL1) involving 72 teeth of which 43 were incisors and canines, and 30 were cheek teeth (Figure 4). Twelve horses (12%) with no history of exodontia had a total of 19 missing teeth. The Triadan 08 and 11 positions were the most affected with seven and nine teeth being absent in these positions, respectively (Figure 5). Two Triadan 10 teeth and one Triadan 03 tooth were also identified as missing.

**Figure 6:**
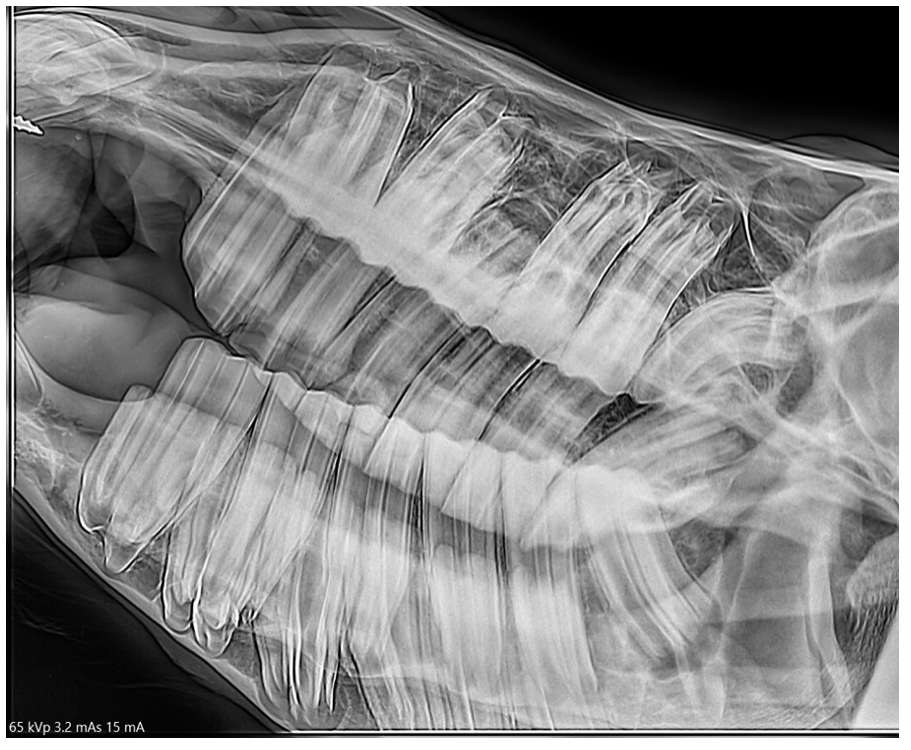
A RtD30-LeVO radiograph of a 4.5 years-old miniature horse depicts a retained 608 and absent 208.

Six horses (6%) had persistent deciduous teeth, including nine incisors and one cheek tooth (Figures 4, 6). Eight dysplastic teeth (5 incisors and 3 cheek teeth) were identified in five horses.

Twenty-one horses (21%) had craniofacial skeletal malocclusions, including 10 with a class 2 maloccusion (overjet/overbite), 10 with a class 3 malocclusion (underjet/underbite), and 1 with a class 4 malocclusion (wry nose).

The less common dental diseases were unerupted teeth, ie dental eruption was more than 6 months past the expected eruption date (4 horses), fractured teeth (2 horses) and supernumerary teeth (1 horse) (13).

### 3.1 Statistical analyses

Spearman correlations found associations between the following patient metrics and dental disease: age in years and diastemata (*p* < 0.001); age in years and # of abnormalities (*p* < 0.001); age in years and number of crown elongations (*p* < 0.001); body weight and presence of class 1 malocclusion (*p* < 0.001); head width and number of abnormalities (*p* = 0.039); head width and presence of class 1 malocclusion (*p* = 0.049).

**Figure 7:**
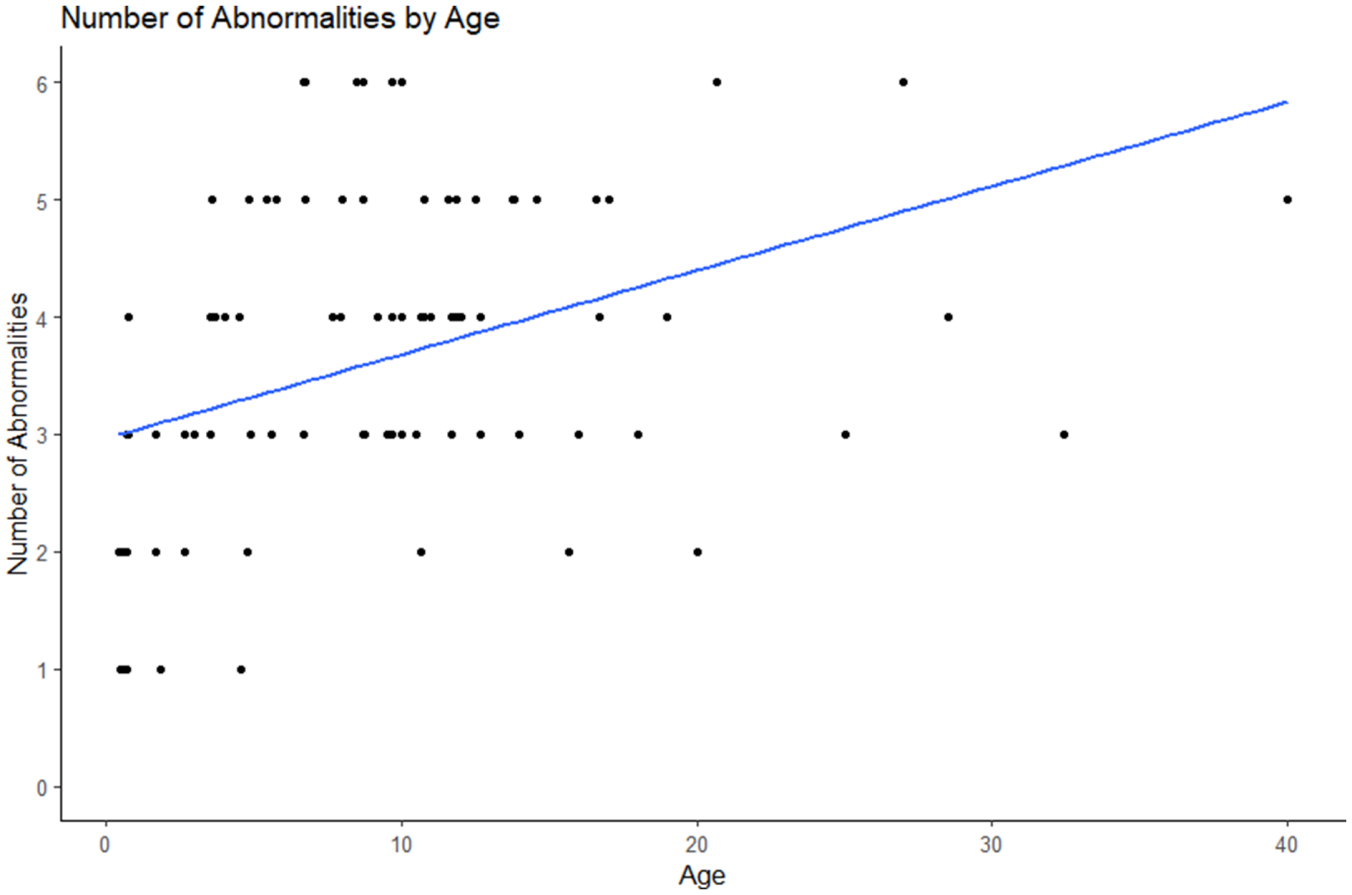
Plot of the number of dental abnormalities vs. the age of the animal. As the age of the animal increased the number of dental abnormalities found per animal increased.

**Figure 8:**
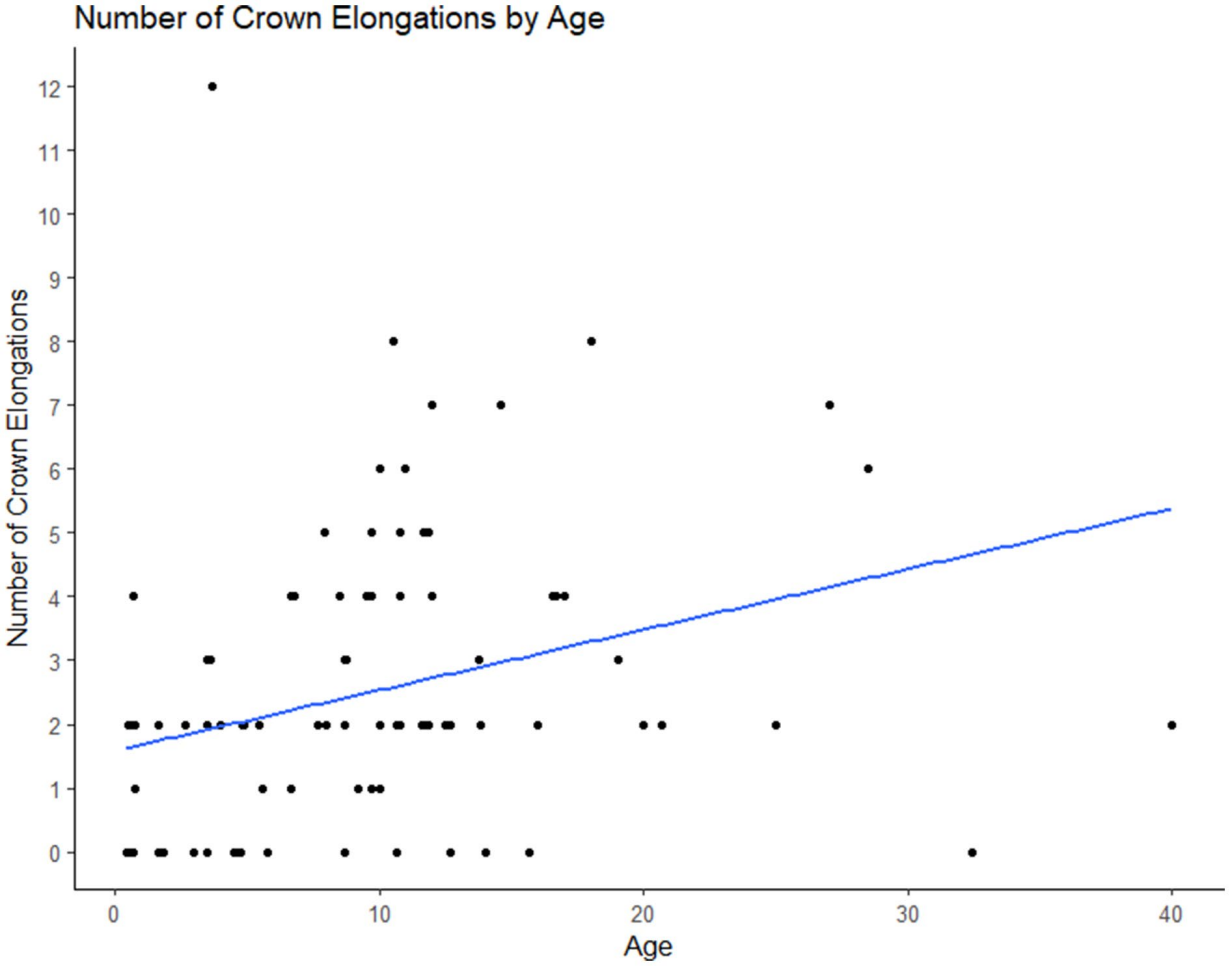
Plot of the number of crown elongations per animal vs. the age of the animal. As the age of the animal increased the number of crown elongations identified also increased.

**Figure 9:**
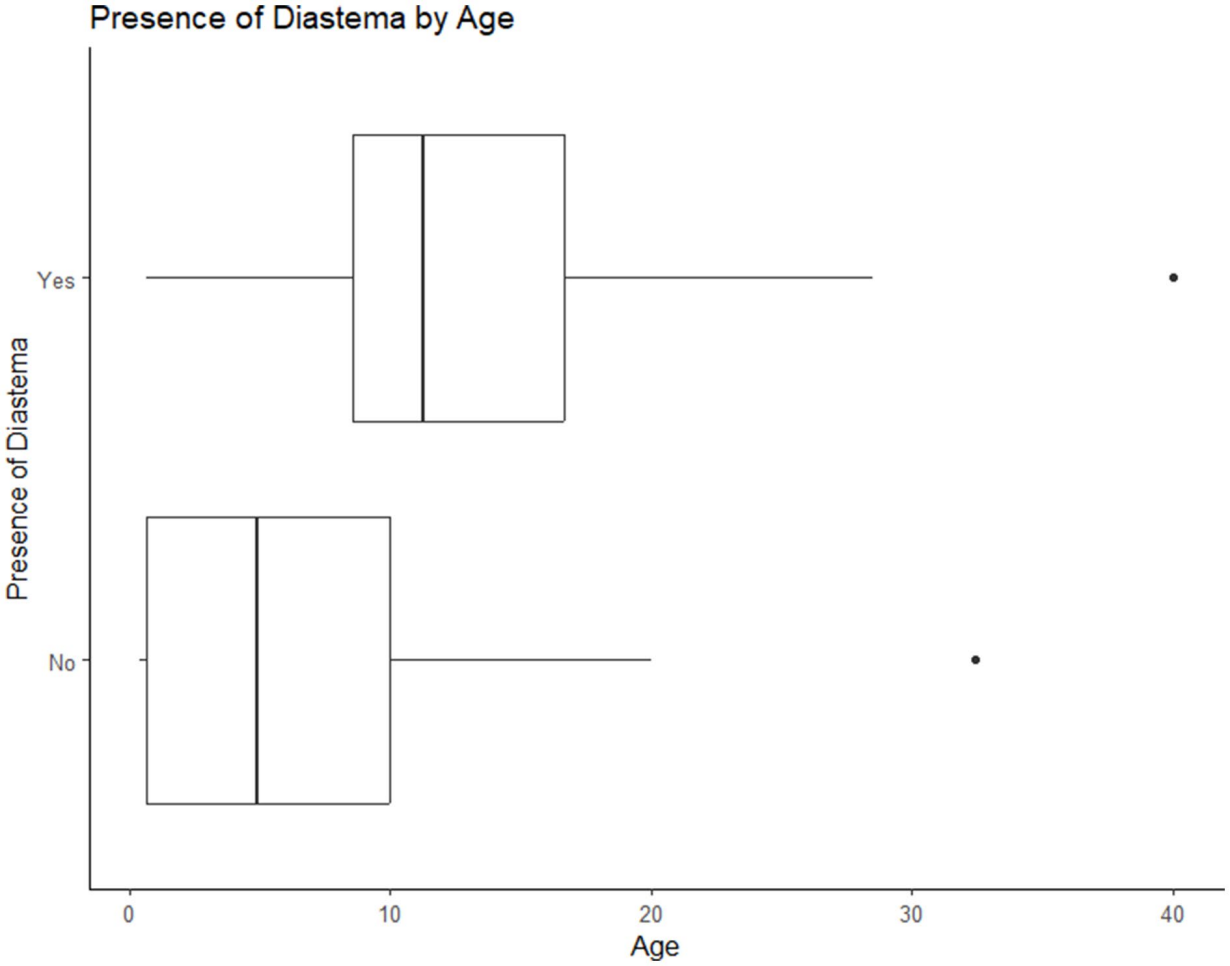
Association of age with diastemata: as the age of the animal increased the likelihood of the presence of diastemata also increased.

The prevalence of abnormalities, diastemata and crown elongations increased with age (Figures 7–9).

**Figure 10:**
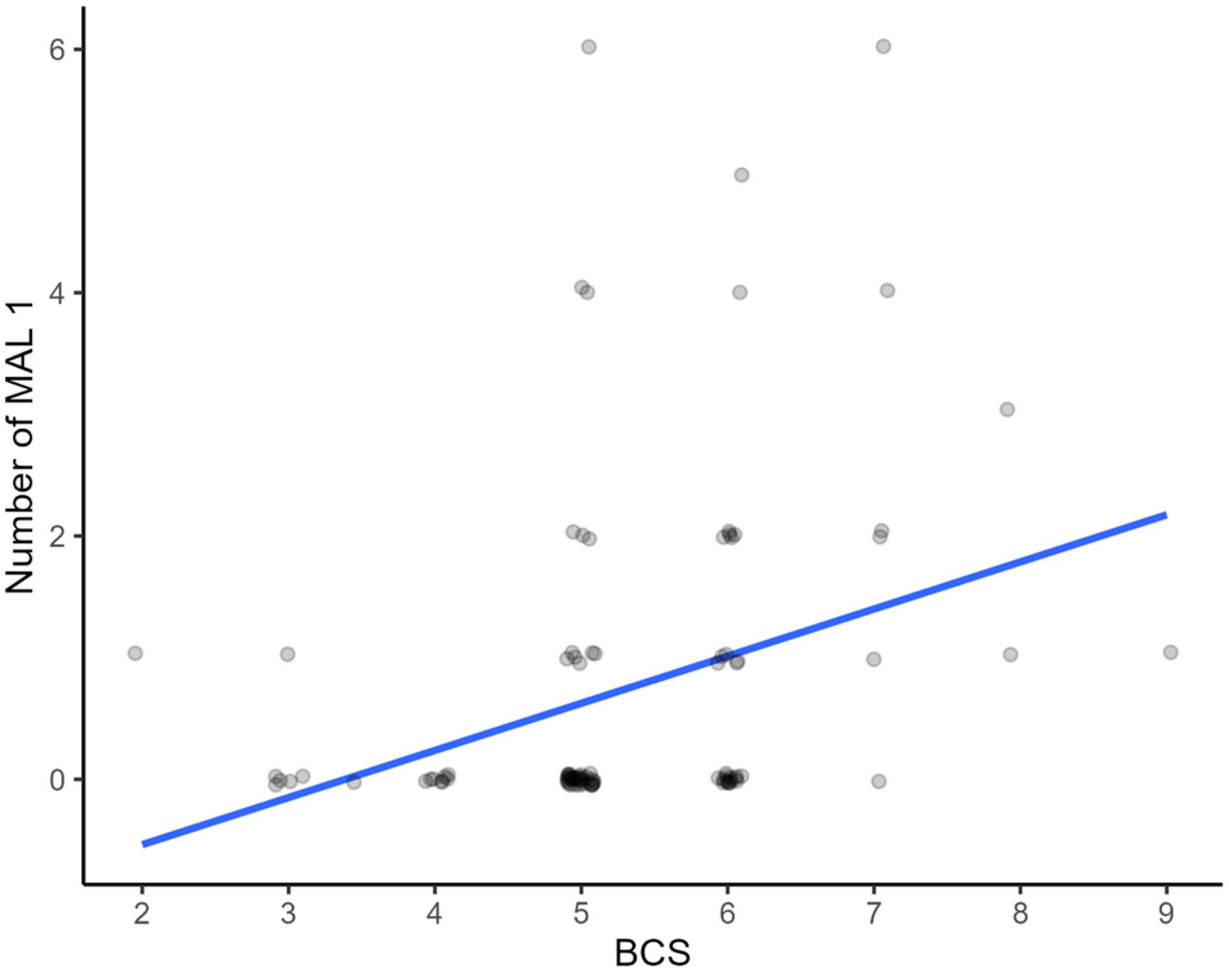
Association of body condition score and class 1 malocclusions (MAL1). Horses with higher body condition scores had a higher prevalence of class 1 malocclusion.

Miniature horses with a high body condition score were significantly more likely to have a class 1 malocclusions (Figure 10).

There was no significant correlation between the length of the head and the presence or number of any category of disease, however the data trended towards increasing numbers of dental abnormalities with increasing head length (*p* = 0.053).

**Figure 11:**
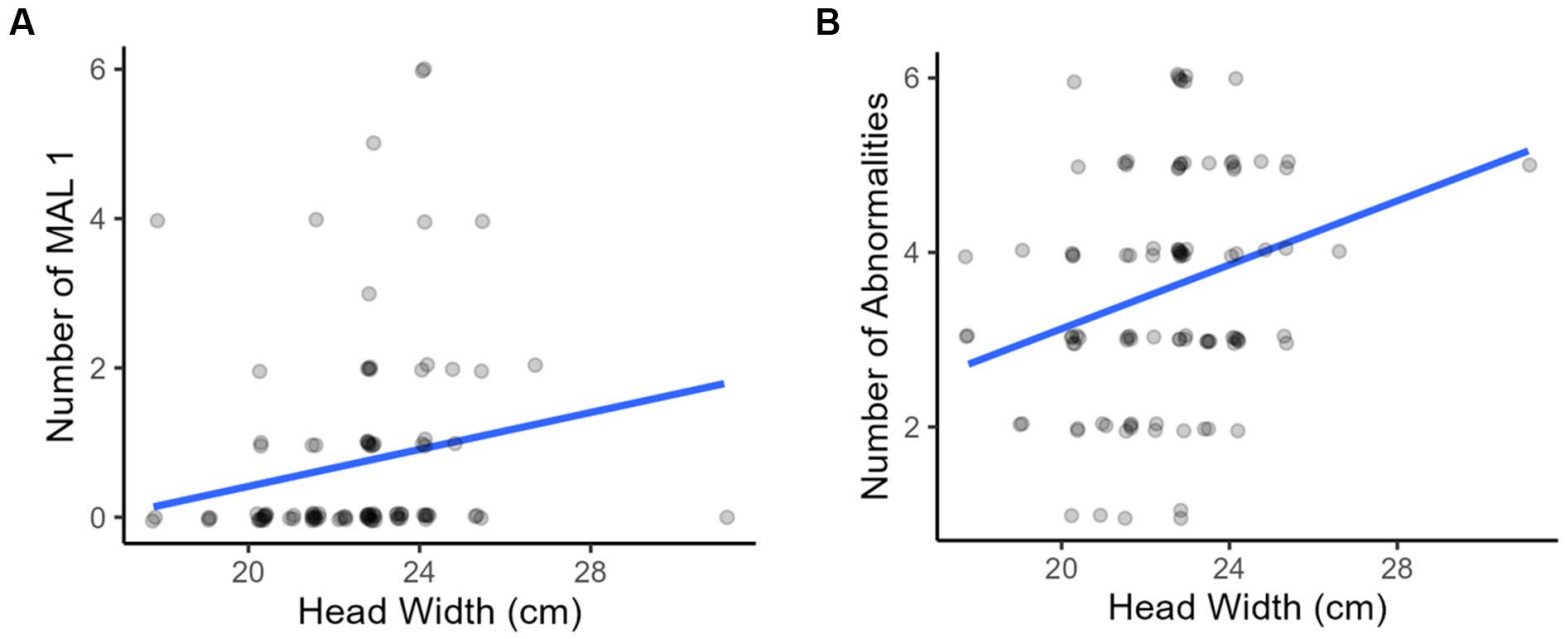
Associations with head width: as the width of the horses’ heads increased there was a **(A)** greater number of class 1 malocclusions and **(B)** greater number of abnormalities on oral examination.

Horses with wider heads were more likely to have a class 1 malocclusions and a higher number of them (Figure 11).

There was no correlation between sex, weight or height at the withers and the presence or frequency of any dental disease.

No significant association was found between the number of elongated crowns and the presence of any class 2 malocclusions (*p* = 0.859) or class 3 malocclusions (*p* = 0.901).

The patterns of teeth affected by a specific disease were analyzed for divergence from a uniform distribution. The results of this analysis are shown in Table 1. The teeth in Triadan positions 01, 02 and 03 (incisors) were more likely to be affected by class 1 malocclusion. Teeth in Triadan positions 08 and 11 were most likely to be missing, and those in Triadan position 06 were most likely to be elongated. A retained deciduous tooth was most associated with the Triadan 03 tooth.

**Table 1:** Breakdown of affected Triadan position by pathology with *p*-values from Monte-Carlo-simulated multinomial testing.

Triadan position	MAL1	Missing tooth	T/U	T/FX	T/EL/CC	T/SN	DT/P	Dysplastic tooth
01	12	0	0	0	1	0	0	0
02	20	0	0	0	3	0	0	2
03	10	1	5	0	7	1	9	3
04	1	0	0	0	0	0	0	0
05	0	0	0	0	0	0	0	0
06	3	0	0	0	111	0	0	0
07	5	0	0	1	7	0	0	0
08	5	7	0	0	32	0	1	0
09	7	0	0	1	15	0	0	1
10	2	2	0	0	15	0	0	1
11	7	9	1	1	49	0	0	1
Estimated *p*-value	*p* = 0.003	*p* = 0.003	*p* = 1	*p* = 1	*p* = 0.003	*p* = 1	*p* = 0.003	*p* = 1

## 4 Discussion

The results of this study confirmed our hypothesis that the prevalence of dental disease among miniature horses is high (95% of miniature horses examined in this study had dental disease). The five most common dental abnormalities in this patient population were crown elongations, oral mucosal ulcerations, diastemata, class 1 malocclusions and hypodontia. Crown elongations (e.g., focal overgrowths, sloping overgrowths, wave mouth, step mouth) (14), oral mucosal ulcerations and diastemata are common dental abnormalities amongst all equids including the miniature horse population (6, 7, 10, 15, 16).

The current MAL1-4 classification system was used in this study to describe occlusion. When interpreting the results, it became apparent that there was a high prevalence of crown elongations at the Triadan 06 and 11 positions with no statistical correlation to the presence/absence of skeletal malocclusion. This information suggests many miniature horses in this population have a mismatch in occlusion of the dental arcades despite matching mandibular and maxillary lengths. It also illustrates that incisor occlusion may not be reflective of cheek teeth occlusion. Alternative reasons for the high number of crown elongations are also considered. These include the high prevalence (12%) of missing teeth and displaced/rotated teeth (MAL1). When a tooth is missing or out of alignment from the opposing arcade an elongation will develop.

The authors identified that the MAL1-4 malocclusion classification system used by the American Veterinary Dental College may not allow for documentation of all types of malocclusion in this population. In future studies we feel it would be prudent to use a subclassification system of the current MAL1-4 designations based on the Ackerman and Profitt classification system used in human orthodontics (17). Horses with neutroclusion of their incisors would be subclassified into MAL1a (mandibular cheek tooth distocclusion) and MAL1b (mandibular cheek tooth mesioclusion). Such a system would more accurately describe dental occlusion patterns in horses with normal skeletal occlusion, i.e., neutroclusion at the incisors and malocclusion at the cheek teeth rows.

Mucosal ulceration was found most commonly on areas of the buccal mucosa that was in contact with the cheek teeth. The presence of enamel points is not considered to be an abnormality, but the soft-tissue damage caused by enamel points was seen frequently in the miniature horse population across all age groups.

Diastemata and class 1 malocclusions were also seen frequently. Diastemata may be primary or acquired (14, 18, 19). We believe the etiologies of diastemata in miniature horses are the same as those of non-miniature horses. The presence of a class 1 malocclusion predisposes to the presence of diastemata which in turn predispose miniature horses to the development of periodontal disease (14, 20).

The prevalence of missing teeth in this population was 12%. The missing teeth were attributed to hypodontia. This diagnosis was based on the radiographic appearance, i.e., absent or reduced dental drift into the vacant alveolus, unlike the situation following extraction (21) as well as history (no known extractions) and the age of the affected animals (Figure 5). Two of the affected horses were >15 years and age-related loss could not be definitively ruled out but was deemed to be unlikely (21). The prevalence of missing teeth in the population of miniature horses we examined was 12%, whereas the prevalence of hypodontia in the overall horse population has been reported at 1% (19, 22). Hypodontia of human beings is defined as a developmental absence of one to five teeth. The terms hypodontia, congenitally missing teeth and agenesis are used interchangeably in the literature on this topic (23). Its aetiogenesis in human beings is known to be multifactorial. Proposed theories include a combination of genetic, epigenetic, environmental, evolutional and anatomical factors (23, 24). In addition, there are multiple systemic syndromes that result in hypodontia including Down Syndrome, Ehlers Danlos syndrome, Fraser syndrome, cleft lip and palate and anhidrotic ectodermal dysplasia (23). To the authors’ knowledge, no studies have been done to determine the etiology of hypodontia in horses, but it is assumed to be the result of similar factors. The authors theorize one anatomical factor for its presence in miniature horses is that the proportionately large teeth and small skulls result in impingement of developing dental buds. This is supported by the fact that the last two teeth to develop (Triadan 08 and 11) were the most frequently affected. Genetic factors may also play a role in development of hypodontia in miniature horses. Registration papers were used to confirm that affected individuals did not share a sire or dam, but further genetic analysis was not performed.

Thirty-four percent of the population was determined to have one or more misaligned teeth (MAL 1), which is a developmental or acquired malocclusion of a specific tooth in relation to the rest of the teeth in the arcade. Types of MAL 1 include labioversion, buccoversion, lingoversion and rotation of a tooth on its longitudinal axis (19, 22). Of the 72 maloccluded teeth, 42 were incisors including 34 in the Triadan 01 and 02 positions. The authors propose that the etiology of this malocclusion is crowding of the teeth caused by an increase in size of the teeth in relation to the size of the jaw (4), a characteristic of the breed resulting from selection for horses small in stature, with a domed head and a refined (small/narrow) muzzle (1). Although displacement of a tooth alone does not create morbidity for the horse, it predisposes the horse to secondary periodontal disease, which in turn may result in pain and/or loss of teeth from periodontal disease (18, 20).

Our statistical analyses showed that as age increases so does the prevalence of diastemata, crown elongations and the total number of dental abnormalities. This correlation between age and the prevalence of dental abnormalities has been reported to occur in non-miniature horses and is proposed to be due to worsening of abnormalities of wear and normal anatomic changes as horses age (18, 20).

In this study horses with higher body condition scores were found to have a higher likelihood for the presence of dental disease. Loss of body condition is often considered a symptom of dental disease (19, 25), but the results of this study provide evidence that loss of body condition should not be used as a benchmark for the presence of dental disease in the miniature horse. Seemingly healthy horses require regular oral examinations to identify the presence of dental diseases.

Head length and head width were measured in this study because of anecdotal reports of dental crowding and proportionally larger teeth. This study showed a significant correlation with increasing head width and the total number of class 1 malocclusions (displaced teeth). These class 1 malocclusions were primarily found in the incisor teeth. Muzzle size and the dimensions of the incisive bone were not measured in this study. It is theorized that although the head is longer the muzzle itself may be smaller in these horses. Additional studies looking at more animals, muzzle dimensions, measurements of the dental arcades and degree of curvature of the arcades may be informative.

There were no direct controls for non-miniature horses in this study so direct comparisons of prevalence could not be made. Data are available in both first opinion and referral populations for both horses and donkeys. There is a wide range of reported prevalence of dental disease reported in equids. Existing surveys estimate it is present in up to 80% (6, 8, 14, 18). The prevalence in this study was 95% showing that dental disease is a highly prevalent condition in miniature horses as it is in their non-miniature counterparts.

In this study a 74% prevalence of crown elongations was found which is notably higher than the 2%–12% prevalence previously reported in donkeys and horses (14, 26). This higher prevalence is theorized to be a result of a large number of miniature horses with rostral or caudal positioning of the maxilla and upper cheek teeth rows, a higher prevalence of craniofacial skeletal malocclusion and of missing teeth. There is little data on the prevalence of class 3 and 4 malocclusions in larger breeds of horses, but the prevalence of class 2 malocclusion has been documented at 2%–5% (27, 28). The 12% prevalence noted in this survey is 2–4× this value. The high prevalence of hypodontia (12%) is 10 times the prevalence reported in the non-miniature population where it has been reported to be the least common developmental dental abnormality in horses (1%) (19, 22). Examination of a larger population is warranted to further investigate these comparisons.

The importance of routine, detailed oral examinations to identify developmental and acquired dental diseases should be emphasized as a part of preventative health care for miniature horses.

## 5 Conclusion

Little published information about dental disease in miniature horses is available. This study illustrates that miniature horses have a high prevalence of dental disease and that they are predisposed to several types of dental disease including malocclusions, hypodontia and crown elongations. Studies are needed to investigate these risk factors for these dental disorders in a larger population of miniature horses. Detailed cephalometric measurements of head size would likely yield more information on risk factors for dental disease. Many of the diseases identified (skeletal and dental malocclusion, hypodontia, retained deciduous teeth) are developmental in nature. It is recommended that early and frequent oral examinations be included as a part of the preventative health program for miniature horses. These examinations can identify the presence of dental diseases and facilitate their management.

## Data availability statement

The original contributions presented in the study are included in the article/Supplementary material, further inquiries can be directed to the corresponding author.

## Ethics statement

The requirement of ethical approval was waived by North Carolina State University College of Veterinary Medicine Institutional Animal Care and Use Committee for the studies involving animals because all procedures being performed were observational and covered under the patient client relationship. The studies were conducted in accordance with the local legislation and institutional requirements. Written informed consent was obtained from the owners for the participation of their animals in this study.

## Author contributions

TT was the primary author and designed the study. TT and EM performed the clinical examinations. CF and EM assisted with data analysis and manuscript preparation. JR performed statistical analyses. All authors contributed to the article and approved the submitted version.

## Footnotes

^1^Equine weight tape: Purina Animal Nutrition (Arden Hills, MN).

^2^XylaMed^™^: Bimeda (Dublin, Ireland).

^3^Dormosedan^®^: Zoetis US (Parsippany-Troy Hills, NJ).

^4^Dolorex^®^: Merck (Millsboro, DE).

^5^Karl Storz Veterinary Endoscopy (Goleta, CA).

^6^Back-Bone Gear Inc.: (Kanata, Ontario Canada).

^7^Cuattro Slate. Heska Corp. (Loveland, CO).

## References

[1] American Miniature Horse Association. About the breed. (2020). Available at: https://www.amha.org/breed-standards. (Accessed September 3, 2023)

[2] American Shetland Pony Club. AMHR. (2021). Available at: https://www.shetlandminiature.com/amhr. (Accessed September 3, 2023)

[3] Mitz C Allen T. “Dentistry in miniature horses,” in Manual of equine dentistry. Ed. Allen T.. Mosby: Maryland Heights, MO (2003). 175–91.

[4] Clauss M Heck L Veitschegger K Geiger M. Teeth out of proportion: smaller horse and cattle breeds have comparatively larger teeth. J Exp Zool B. (2022) 338:561–74. doi: 10.1002/jez.b.23128, PMID: 35286773 PMC9790632

[5] Heck L Sanchez-Villagra MR Stange M. Why the long face? Comparative shape analysis of miniature, pony, and other horse skulls reveals changes in ontogenetic growth. PeerJ. (2019) 7:e7678. doi: 10.7717/peers.7678, PMID: 31576240 PMC6752190

[6] du Toit N Kempson SA Dixon PM. Clinical dental examinations of 357 donkeys in the UK. Part 1. Equine Vet J. (2009) 41:390–4. doi: 10.2746/042516409x368912, PMID: 19562902

[7] Górski K Stefanik E Turek B Bereznowski A Czopowicz M Polkowska I. Malocclusions and dental diseases in privately owned horses in the Mazovia region of Poland. Animals. (2022) 12:3120. doi: 10.3390/ani12223120, PMID: 36428347 PMC9686654

[8] Anthony J Waldner C Grier C Laycock AR. A survey of equine oral pathology. J Vet Dent. (2010) 27:12–5. doi: 10.1177/089875641002700102, PMID: 20469790

[9] Samad L Tavanaeimanesh H Mehr Azin HM Moadab SH Vajhi AR. Clinical dental finding in Iranian horses. Vet Med Sci. (2020) 6:679–85. doi: 10.1002/vms3.329, PMID: 32735069 PMC7738718

[10] Vemming DC Steenkamp G Carstens A Olorunju SAS Stroehle RM Page PC. Prevalence of dental disorders in an abattoir population of horses in South Africa by oral examination of intact and bisected heads. Vet J. (2015) 205:110–2. doi: 10.1016/j.tvjl.2015.03.021, PMID: 25979819

[11] American Veterinary Dental College. AVDC abbreviations. (2023) Available at: https://avdc.org/resident-services/. (Accessed September 3, 2023).

[12] R Core Team. R: a language and environment for statistical computing. Vienna, Austria: R Foundation for Statistical Computing (2022) Available at: https://www.r-project.org.

[13] Muylle S Simoens P Lauwers H Van Loon G. Age determination in mini-Shetland ponies and donkeys. J Vet Med A. (1999) 46:421–9. doi: 10.1046/j.1439-0442.1999.00229.x, PMID: 10528536

[14] Dixon PM du Toit N Borkent D. “Equine dental pathology”. In: Easley J Dixon PM Toit N du, editors. Equine dentistry and maxillofacial surgery. Newcastle upon Tyne: Cambridge Scholars Publishing (2022). p. 161–197.

[15] du Toit N Burden FA Dixon PM. Clinical dental findings in 203 working donkeys in Mexico. Vet J. (2008) 178:380–6. doi: 10.1016/j.tvjl.2008.09.013, PMID: 18977674

[16] Chinkangsadarn T Wilson GJ Greer RM Pollitt CC Bird PS. An abattoir survey of equine dental abnormalities in Queensland, Australia. Aust Vet J. (2015) 93:189–94. doi: 10.1111/avj.12327, PMID: 26010923

[17] Ghodasra R Brizuela M. Orthodontics, malocclusion. Treasure Island (FL): StatPearls Publishing, LLC (2023).

[18] Nuttall HE Ravenhill PJ. Prevalence and analysis of equine periodontal disease, diastemata and peripheral caries in a first-opinion horse population in the UK. Vet J. (2019) 246:98–102. doi: 10.1016/j.tvjl.2019.02.005, PMID: 30902197

[19] Dixon PM Tremaine WH Pickles K Kuhns L Hawe C McCann J. Equine dental disease part 2: a long-term study of 400 cases: disorders of development and eruption and variations in position of the cheek teeth. Equine Vet J. (1999) 31:519–28. doi: 10.1111/j.2042-3306.1999.tb03862.x, PMID: 10596936

[20] Collins NM Dixon PM. Diagnosis and management of equine diastemata. Clin Tech Equine Pract. (2005) 4:148–54. doi: 10.1053/j.ctep.2005.04.006

[21] Giavitto AE Meehan LJ. Congenital hypodontia in three horses diagnosed by computed tomography. Equine Vet Educ. (2020) 33:352. doi: 10.1111/eve.13270

[22] Dixon PM Vlaminck L. Abnormalities of craniofacial development and of dental development and eruption In: Easley J Dixon P du Toit N, editors. Equine dentistry and maxillofacial surgery. Newcastle upon Tyne (UK): Cambridge Scholars Publishing (2022). 141–60.

[23] Meade MJ Dreyer CW. Tooth agenesis: an overview of diagnosis, aetiology and management. Jpn Dent Sci Rev. (2023) 59:209–18. doi: 10.1016/j.jdsr.2023.07.001, PMID: 37645267 PMC10461125

[24] Khalaf K Miskelly J Voge E Macfarlane TV. Prevalence of hypodontia and associated factors: a systematic review and meta-analysis. J Orthod. (2014) 41:299–316. doi: 10.1179/1465313314Y.0000000116, PMID: 25404667

[25] Pehkonen J Karma L Raekallio M. Behavioral signs associated with equine periapical infection in cheek teeth. J Equine Vet. (2019) 77:144–50. doi: 10.1016/j.jevs.2019.03.005, PMID: 31133309

[26] du Toit N Burden FA Dixon PM. Clinical dental examinations of 357 donkeys in the UK. Part 2: epidemiological studies on the potential relationships between different dental disorders, and between dental disease and systemic disorders. Equine Vet J. (2009) 41:395–400. doi: 10.2746/042516409x368903, PMID: 19562903

[27] Domanska-Kruppa N Venner M Bienert-Zeit A. Cephalometric study of the overjet development in warmblood foals. Front Vet Sci. (2019) 6:431. doi: 10.3389/fvets.2019.00431, PMID: 31850386 PMC6895015

[28] Uhlinger C.. Survey of selected dental abnormalities in 233 horses. Proceedings of the 33rd Annual Meeting of the Association of Equine Practitioners (1987) 577–583.

