# Supplementary material for: The prevalence and risk factors of dental disease found in 100 miniature horses
Source: Front Vet Sci. 2023 Nov 30;10:1239809. doi: 10.3389/fvets.2023.1239809 (PMC10720032; doi:10.3389/fvets.2023.1239809)
Supplement: Supplementary file 1 [file Data_Sheet_1.docx]

Supplementary Material

The Incidence and Risk Factors of Dental Disease Found in 100 Miniature Horses

Tracy Tinsley*^1^, Callie Fogle^2^, Elaine Means^3^, James Robertston^4^

^1^Elite Equine Mobile Dentistry, Holly Springs, NC, USA

^2^Department of Clinical Sciences, North Carolina State University College of Veterinary Medicine, Raleigh, NC, USA

^3^Elaine Means Mobile Equine Dentistry, Sanford, NC USA

^4^Office of Research, North Carolina State University College of Veterinary Medicine, Raleigh, NC USA

*** Correspondence:** Tracy Tinsley tracytinsleydvm@gmail.com

## Supplementary Tables:

Supplementary Table 1: Patient metrics

|  | Age (years) | Body Weight (kg) | Body Condition Score (1-9) | Wither height (cm) | Head Length (cm) | Head Width (cm) |
| --- | --- | --- | --- | --- | --- | --- |
| Range | .42-40 | 37.37-177.3 | 2-9 | 73.66-111.76 | 31.75-43.18 | 17.78-26.67 |
| Median | 8.56 | 122.50 | 5 | 93.98 | 37.8 | 22.86 |
| Mean | 8.58 | 118.22 | 5.25 | 92.97 | 37.24 | 22.48 |
| Standard deviation | 7.41 | 33.31 | 1.11 | 9.36 | 3.0 | 1.95 |

Supplementary Table 2: P-values for association of pathology with patient metrics: (* included for p<0.05)

| Measure | Sex | Age in years | Weight kgs | BCS 1-9 | Wither height cm | Head length cm | Head Width cm |
| --- | --- | --- | --- | --- | --- | --- | --- |
| Diastema | p=0.149 (wilco) | p<0.001* (No Diastema: 6.38 [0.67, 10],  Diastema: 12.87 [8.54, 16.65]) | p=1 (wilco) | p=1 (wilco) | p=1 (wilco) | p=1 (wilco) | p=1 (wilco) |
| C/L | p=1 (wilco) | p=1 (wilco) | p=1 (wilco) | p=1 (wilco) | p=1 (wilco) | p=1 (wilco) | p=1 (wilco) |
| # of complication | p=1 (cor) | p<0.001* (r=0.506) | p=1 (cor) | p=0.117 (cor) | p=0.919 (cor) | p=0.053 (cor) | P=0.039* (r=0.276) |
| Any complication | All responses 1 | All responses 1 | All responses 1 | All responses 1 | All responses 1 | All responses 1 | All responses 1 |
| Other MAL (MAL 2, 3 or 4) | p=0.798 (wilco) | p=1 (wilco) | p=1 (wilco) | p=1 (wilco) | p=1 (wilco) | p=0.248 (wilco) | p=0.095 (wilco) |
| MAL 1 | p=1 (cor) | p=0.092 (cor) | p=1 (cor) | p<0.001* (r=0.393) | p=1 (cor) | p=1 (cor) | p=0.049* (r=0.268) |
| No. Missing Tooth | p=1 (cor) | p=0.387 (cor) | p=1 (cor) | p=1 (cor) | p=1 (cor) | p=1 (cor) | p=1 (cor) |
| No. T/U | p=1 (cor) | p=1 (cor) | p=1 (cor) | p=1 (cor) | p=1 (cor) | p=1 (cor) | p=1 (cor) |
| No. T/FX | p=1 (cor) | p=1 (cor) | p=1 (cor) | p=1 (cor) | p=1 (cor) | p=1 (cor) | p=1 (cor) |
| No. T/EL/CC | p=1 (cor) | p<0.001* (r=0.438) | p=1 (cor) | p=1 (cor) | p=1 (cor) | p=1 (cor) | p=1 (cor) |
| No. T/SN | p=1 (wilco) | p=1 (wilco) | p=1 (wilco) | p=1 (wilco) | p=1 (wilco) | p=1 (wilco) | p=1 (wilco) |
| No. DT/P | p=1 (cor) | p=1 (cor) | p=1 (cor) | p=1 (cor) | p=1 (cor) | p=1 (cor) | p=1 (cor) |
| No. Dysplastic teeth | p=1 (cor) | p=0.068 (cor) | p=1 (cor) | p=1 (cor) | p=1 (cor) | p=1 (cor) | p=1 (cor) |
| Cheek teeth MAL 1 | p=1 (cor) | p=0.264 (cor) | p=1 (cor) | p=0.292 (cor) | p=0.479 (cor) | p=1 (cor) | p=1 (cor) |
| Cheek teeth – missing Tooth | p=1 (cor) | p=0.706 (cor) | p=1 (cor) | p=1 (cor) | p=1 (cor) | p=1 (cor) | p=1 (cor) |
| Cheek teeth – T/U | p=1 (wilco) | p=1 (wilco) | p=1 (wilco) | p=1 (wilco) | p=1 (wilco) | p=1 (wilco) | p=1 (wilco) |
| Cheek teeth – T/FX | p=1 (cor) | p=1 (cor) | p=1 (cor) | p=1 (cor) | p=1 (cor) | p=1 (cor) | p=1 (cor) |
| Cheek teeth – T/EL/CC | p=1 (cor) | P<0.001* (r=0.389) | p=1 (cor) | p=1 (cor) | p=1 (cor) | p=1 (cor) | p=1 (cor) |
| Cheek teeth - T/SN | All responses 0 | All responses 0 | All responses 0 | All responses 0 | All responses 0 | All responses 0 | All responses 0 |
| Cheek teeth - DT/P | p=1 (wilco) | p=1 (wilco) | p=1 (wilco) | p=1 (wilco) | p=1 (wilco) | p=1 (wilco) | p=1 (wilco) |

**
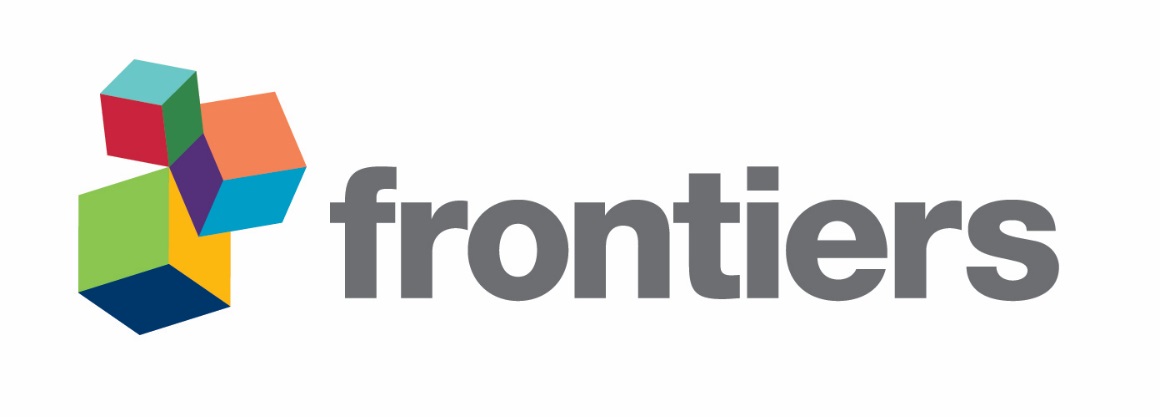
**
